# Atomic-Scale Understanding on the Tribological Behavior of Amorphous Carbon Films under Different Contact Pressures and Surface Textured Shapes

**DOI:** 10.3390/ma16186108

**Published:** 2023-09-07

**Authors:** Zan Chen, Naizhou Du, Xiaowei Li, Xubing Wei, Jiaqing Ding, Shiqi Lu, Shuangjiang Du, Cunao Feng, Kai Chen, Dekun Zhang, Kwang-Ryeol Lee

**Affiliations:** 1 School of Materials Science and Physics, China University of Mining and Technology, Xuzhou 221116, China; ts22180025a31@cumt.edu.cn (Z.C.); dnz1025@163.com (N.D.); xbwei@cumt.edu.cn (X.W.); dingjiaqing0325@163.com (J.D.); lushiqi9809@163.com (S.L.); m13551419671@163.com (S.D.); cunaofeng@cumt.edu.cn (C.F.); cumtck@cumt.edu.cn (K.C.); dkzhang@cumt.edu.cn (D.Z.); 2 Key Laboratory of Marine Materials and Related Technologies, Zhejiang Key Laboratory of Marine Materials and Protective Technologies, Ningbo Institute of Materials Technology and Engineering, Chinese Academy of Sciences, Ningbo 315201, China; 3 Computational Science Center, Korea Institute of Science and Technology, Seoul 136-791, Republic of Korea

**Keywords:** amorphous carbon, friction property, surface texture, reactive molecular dynamics

## Abstract

The textured design of amorphous carbon (a-C) film can significantly improve the tribological performance and service life of moving mechanical components. However, its friction dependence on different texture shapes, especially under different load conditions, remains unclear. In particular, due to the lack of information regarding the friction interface, the underlying friction mechanism has still not been unveiled. Therefore, the effects of contact pressure and textured shapes on the tribological behavior of a-C films under dry friction conditions were comparatively studied in this work by reactive molecular dynamics simulation. The results show that under low contact pressure, the tribological property of a-C film is sensitive to the textured shape, and the system with a circular textured surface exhibits a lower friction coefficient than that with a rectangular textured surface, which is attributed to the small fraction of unsaturated bonds. However, the increase of contact pressure results in the serious reconstruction and passivation of the friction interface. On the one hand, this induces a growth rate of friction force that is much smaller than that of the normal load, which is followed by a significant decrease in the friction coefficient with contact pressure. On the other hand, the destruction or even disappearance of the textured structure occurs, weakening the difference in the friction coefficient caused by different textured shapes of the a-C surface. These results reveal the friction mechanism of textured a-C film and provide a new way to functionalize the a-C as a protective film for applications in hard disks, MEMS, and NEMS.

## 1. Introduction

Due to the friction and wear of mechanical moving parts, a great quantity of energy dissipation and material loss are caused. Therefore, to increase the effectiveness and resistance to wear and friction of mechanical systems, such as aerospace, automotive, and microelectromechanical systems, it is required that one create cutting-edge lubricating materials and technologies [1,2]. As the structure of the controlled nanocomposite, amorphous carbon (a-C) film, consisting of sp^3^, sp^2^, and sp bonding states, is endowed with exceptional physicochemical properties, such as corrosion resistance, a low friction coefficient, high hardness, good resistance to wear, biocompatibility, etc. [3,4,5,6]. It has become a strong candidate as a protective film to improve the tribological properties of mechanical parts [7,8].

In the past decades, relevant researchers have looked into how different variables affect how a-C films behave when it comes to friction, including elemental dopants, sliding velocity, applied load, environmental conditions, etc. For example, Li [9] investigated the friction properties of self-mated a-C films with hydrogenated surfaces through reactive molecular dynamics (RMD) simulation and reported that the hydrogenated treatment of an a-C surface significantly improved tribological properties under low load conditions without impairing the intrinsic properties of a-C film, which was attributed to the hydrogen-induced repulsive effect and interfacial passivation. Wei [10] studied the influence of sliding speeds on the friction behavior of a-C film, and the results showed that the development of friction with sliding time had two modes: sawtooth-type stick-slip friction at a low sliding speed and smooth friction at a high sliding speed. Wang [11] systemically discussed the effects of load on the tribological behaviors of a-C film and reported that both the friction coefficient and wear rate of a-C film fell off as the applied load rose, with the friction coefficient being mainly dependent on the graphitization level of the interface, while both the decreased friction coefficient and increased heat dissipation contributed to the decrease of the wear rate.

In particular, texturing the a-C surface to improve its friction-reducing performance has recently attracted widespread attention [12]. The textured surface of a-C films facilitates a decrease in the contact surface area within the friction interface and also reduces the level of interconnection between the corresponding materials. Furthermore, the overall friction efficiency is synergistically enhanced through the retention of graphitized abrasive particles while sliding [13,14]. For example, Du [15] studied the relationship of the friction behavior of a-C film with the textured parameters under oil lubrication and disclosed that the friction coefficient first decreased as the textured depth grew before increasing again, and the lubrication mechanism also changed from fluid lubrication to boundary lubrication. Arslan et al. [16] observed that when the textured structure was designed with ideal dimensions (100 μm in diameter and 6 μm in depth), the friction coefficient of the a-C film significantly increased with the texture’s size.

It is commonly accepted that the surface makeup of mating materials has a complex relationship with the friction and wear of a-C films. Many textured configurations of the a-C surface produce a variety of interfacial structures, as well as increasing the complexity and multiplicity of the friction interface [17,18]. Although the influence of surface texture on the tribological properties of a-C films has been studied, its dependence on different textured shapes, especially under different load conditions that have a significant effect on the interaction between mating films, remains unclear. In addition, due to the limitation of experimental characterization, a thorough understanding of how surface texturing affects tribological characteristics of a-C films at an atomic/electronic scale, particularly the discernment of information regarding the friction interfaces, is still lacking. As a result, the underlying friction mechanism, as well as the efficiency and selectivity of the surface texture design of a-C films, are left without a clear explanation.

In this work, the different textured shapes of a-C surfaces, including circle and rectangle, were fabricated separately. The tribological behavior and interfacial structure transformation of a-C films with various surface’s textured shapes were studied comparatively using RMD simulations. In particular, their friction dependence on different contact pressures was also considered. These results can provide theoretical guidance for designing the textured structure of a-C surfaces with a high performance for anti-friction applications.

## 2. Methods

**Figure 1:**
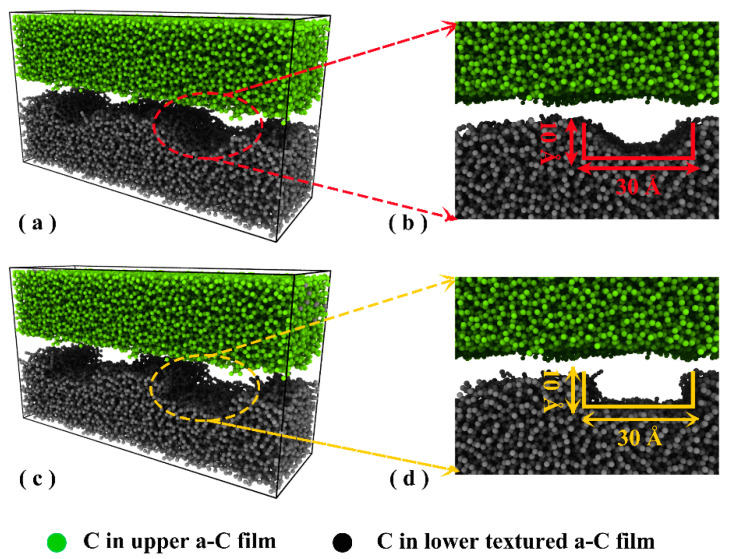
Friction model composed of a-C films with different textured shapes as bottom layers and untextured a-C film as an upper layer: (**a**,**b**) Circle-textured shape and its textured parameters; (**c**,**d**) rectangle-textured shape and its textured parameters.

RMD simulations were conducted by a Large-scale Atomic/Molecular Massively Parallel Simulator (LAMMPS) [19] to explore the friction process of a-C films. The friction model is depicted in Figure 1 and was composed of an untextured upper layer and a lower film with texture. First, the preliminary a-C structure was directly created as the top film using an atom-by-atom deposition simulation with a kinetic energy of 70 eV/atom per incident C atom and was applied as the upper layer directly. Then, its surface was further tailored into different textured shapes, including circle and rectangle, as the lower textured layer in Figure 1, respectively [20]. For the friction model with the circular textured a-C (Figure 1a), its dimensions were 128.64 × 40.36 × 71.63 Å^3^ with 39,753 carbon atoms. For the case with rectangular textured a-C (Figure 1c), its dimensions were 128.64 × 40.36 × 71.89 Å^3^ with 38,913 carbon atoms. The textured parameters for each case are provided in Figure 1b,d, respectively. Because of the limitations of the RMD simulation, the nanoscale sizes of the textured parameters were considerably smaller than those seen in the experiment [12,21]. Nonetheless, these size restrictions proved sufficient for extracting the crucial information regarding the friction interface in situ and providing valuable insights into the fundamental disparities arising from the design of the a-C surface’s texture. This, in turn, aids in guiding the design of a-C films for various applications, such as MEMS, hard-disk technology, and so on [22,23].

The upper and bottom a-C layers were split into a fixed layer, a thermostatic layer, and a free layer, respectively, prior to the friction process. The detail was shown in a previous study [15], in which the fixed layer simulated the infinite system, the thermostatic layer was kept at 300 K, while the free later simulated the complicated structural transformation of the friction interface. During the friction process, the system first relaxed for 25 ps at 300 K before the friction process began, and then the normal load was applied to the fixed layer of the upper a-C film until the specified contact pressure value (5, 20, 50 GPa) was reached; finally, the upper a-C fixed layer slid along the *x* direction to simulate the friction shearing process. The sliding speed was 100 m/s, and the sliding time was 750 ps. The C-C interaction was described using the reactive force field potential developed by Tavazza [24], the time step was 0.25 fs, and both the *x* and *y* directions were applied to the periodic boundary conditions. According to the different contact pressures (5, 20, 50 GPa) and textured shapes (circle, rectangle), the friction systems were separately named a-C@C5, a-C@C20, a-C@C50, a-C@R5, a-C@R20, and a-C@R50.

## 3. Results and Discussion

**Figure 2:**
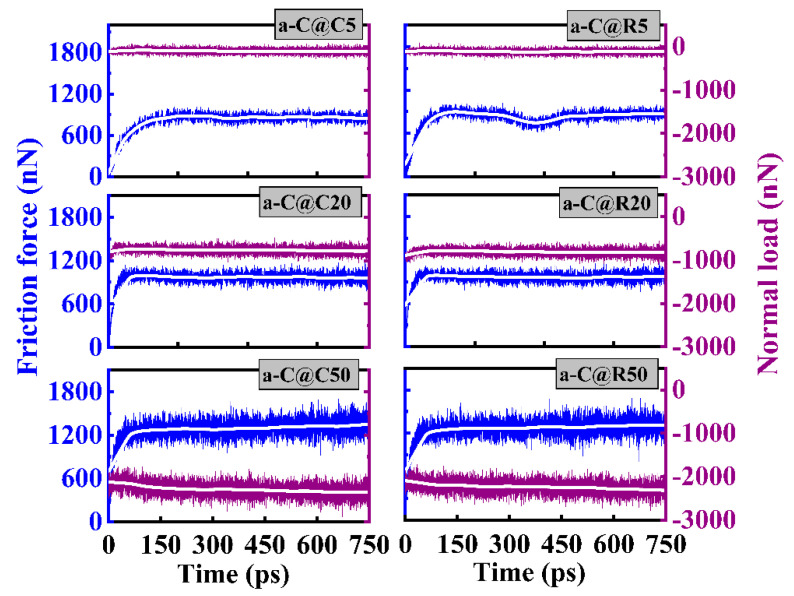
Friction-force and normal-load curves of friction systems with circle- and rectangle-textured shapes, respectively, under different contact pressures.

The friction curves for each circular and rectangular textured system under various contact pressures are shown in Figure 2, along with the normal forces, sliding time, and friction forces. It is obvious for each system that there is an initial increase in friction force before it stabilizes once the sliding process is finished, but the normal load curve changes smoothly with the sliding time, suggesting a slight variation of the real contact area at the friction interface. The significant increase in the friction force corresponds to the running-in process, in which the shearing effect, especially under high pressure, causes a larger deformation, and even collapse, of the textured layer. Meanwhile, the stabilized stage in the friction force suggests that the interface reconstruction is gradually completed.

Hertzian theory describes the relationship between the contact pressure, real contact area, and normal force of the friction system.

(1) $$A=\frac{W}{\sigma }$$

where *W* is the applied normal force, *σ* is the Hertzian contact pressure, and *A* is the real contact area. The results indicate that more C atoms come into touch with one another when the contact pressure rises, increasing the real contact area and the cross-linking degree of systems. Hence, this change in normal force originates from the evolution of the real contact area with the contact pressure (as illustrated in Figure S1 of the Supplementary Data). Additionally, because the rough shape of the low a-C surface is fixed, with the increase of contact pressure, the friction fluctuates in an obvious manner, and the friction process quickly enters into the stage of steady friction, which is comparable to the untextured system cited in the previous study [15]; the normal load also increases gradually with the increase of contact pressure. However, there is no obvious difference between the changes shown in the normal-force and friction-force curves with textured shapes under the same contact pressure. This indicates that the contact pressure, rather than the texture of the a-C surface, has a stronger impact on the tribological behavior of systems.

To compare the tribological properties of a-C systems with the textured shapes and contact pressures, the average friction force, *f*, and normal load, *W*, are obtained using the values located at the last 200 ps of the friction curves in Figure 2, which are then utilized to determine the friction coefficient for each system using the equation that follows [15]:

(2) $$\mu =\frac{f}{W}$$

**Figure 3:**
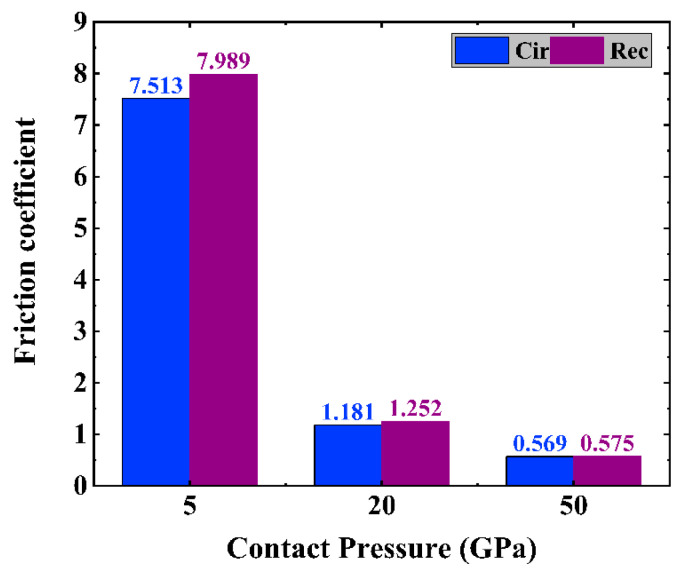
Friction coefficient of friction systems with circle- and rectangle-textured shapes, respectively, under different contact pressures.

The computed μ for each system is shown in Figure 3. The results show that the friction coefficient of the a-C system decreases with the contact pressure under the same textured shape. However, the variation in the friction coefficient with the contact pressure affects how the a-C surface is textured. When there is a 5 GPa contact pressure, the friction coefficient for the system with the circular texture is smaller than that with the rectangular texture; however, if the contact pressure is elevated to 20 and 50 GPa, this difference in the friction coefficient, which is caused by different textured shapes, becomes weak, even disappearing. These results suggest different interfacial structural transformations when friction occurs, as will be discussed in detail later. In addition, it is observed that because surface contamination and passivation are missing, the friction coefficient values in the experiment are frequently greater than the values of the friction coefficient obtained from the present models in Figure 3 [25,26].

**Figure 4:**
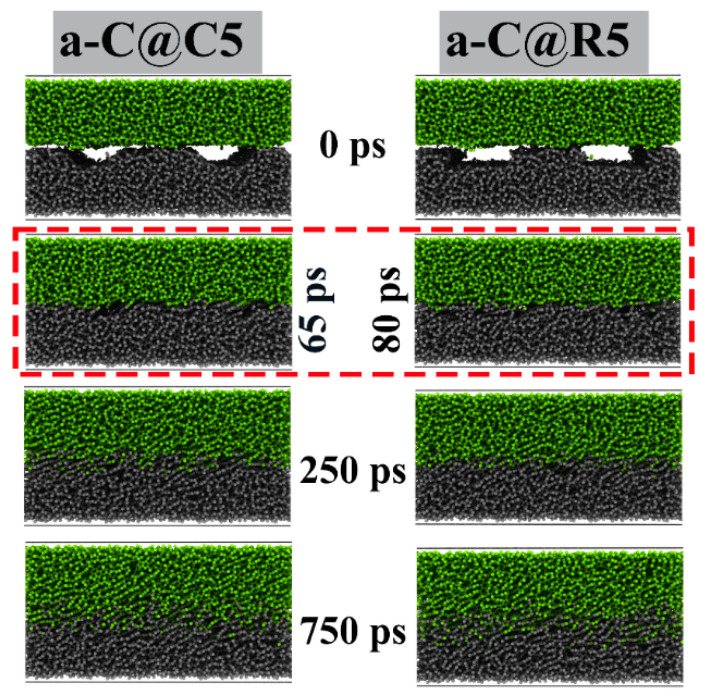
Evolutions of interfacial morphologies of a-C@C5 and a-C@R5 systems, respectively.

For a-C@C5 and a-C@R5 systems, Figure 4 depicts the evolution of interfacial morphologies during the sliding processes. It is worth noting that under dry friction conditions, a significant change in the interface structure is observed with a sliding time ranging from 0 to 750 ps due to the interaction between the upper and lower a-C surfaces. For the a-C@C5 system, the textured layer is worn out at 65 ps, and the interface collapses and restructures, while for the a-C@R5 system, a similar situation occurs at 80 ps; with the further increase of the sliding time, the amount of cross-linking between the upper and lower a-C surfaces gradually rises.

Figure S2 of the Supplementary Data shows the morphologies of the a-C@C50 and a-C@R50 friction systems when the contact pressure is further increased to 50 GPa. Compared with the results under 5 GPa (Figure 4), a rise in contact pressure leads to the collapse of the a-C@C50 and a-C@R50 textured layers at the beginning of the sliding process, and the cross-linking degree of a-C surfaces is also much more serious than that of a-C@C5 and a-C@R5 systems. As a result, the shape of the textured layer has little influence on the tribology of the system at a contact pressure of 50 GPa.

**Figure 5:**
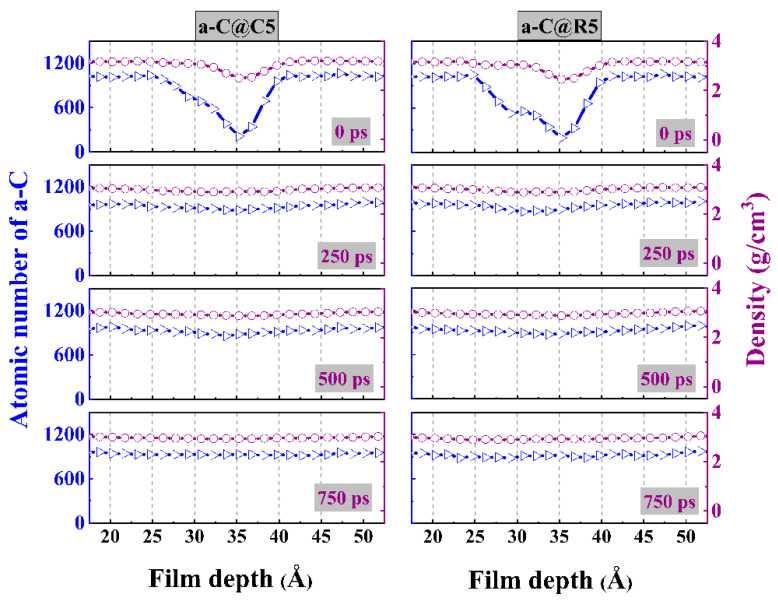
Atomic number and density distributions of a-C@C5 and a-C@R5 friction systems, respectively.

Furthermore, the atomic number and density distributions of a-C@C5 and a-C@R5 systems along the film-depth direction are quantitatively analyzed (Figure 5). Notably, the interfacial structure has a significant impact on the friction behavior of a-C films. Under a contact pressure of 5 GPa, the change in the atomic number and density with the sliding time exhibits a similar trend for these two systems due to the gradual collapse of the interfacial textured layer. This is in line with the findings in Figure 4. However, under a high contact pressure (Figure S3 of the Supplementary Data), although a similar behavior occurs, the characteristic curves for the textured layer are destroyed even at the beginning of the sliding process due to the high-load-induced collapse. This can account for the short running-in process under high contact pressure, as observed in Figure 2.

**Figure 6:**
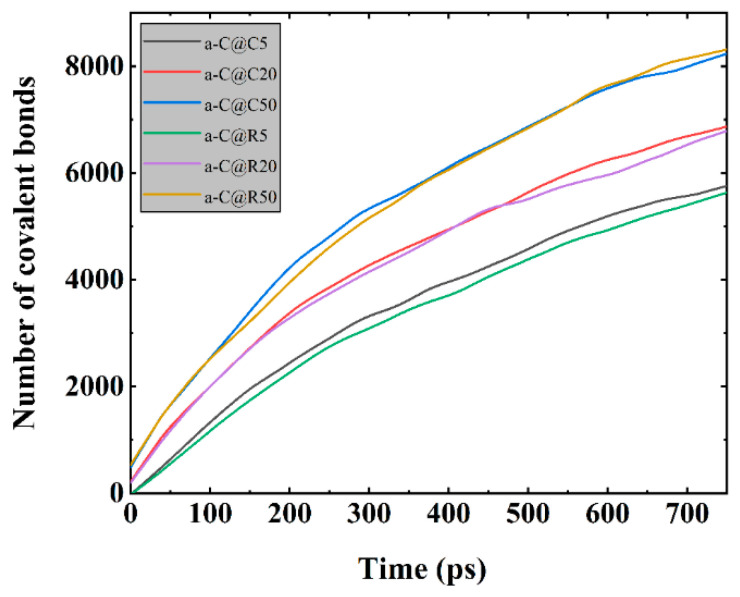
Change in the number of covalent bonds between mating a-C surfaces for friction systems with circle- and rectangle-textured shapes, respectively, under different contact pressures.

To assess how contact pressure and texture affect the tribological properties of a-C film, the comparison and examination of the modifications require the quantity of increased C-C covalent connections between a-C surfaces that are mated due to the cross-linking when sliding is in progress [9,15]. Figure 6 shows that the number of C-C covalent bonds increases steadily with the sliding time for each system. When the contact pressure increases from 5 to 50 GPa, deeper cross-linking between the top and lower a-C surfaces occurs, and thus the number of additional C-C covalent bonds continues to increase. By comparing the a-C@C5 system with the a-C@R5 system, it is found that the increased number of C-C covalent bonds in the a-C@C5 system is greater than that in the a-C@R5 system, indicating that under a contact pressure of 5 GPa, the interfacial collapse of the a-C@C5 system during sliding is faster than that of a-C@R5, as confirmed by Figure 4. As a result, the system enters a smooth friction motion in advance, resulting in an increased number of C-C covalent bonds that is higher than for a-C@R5, which is similar to the results of Du et al. [15].

**Figure 7:**
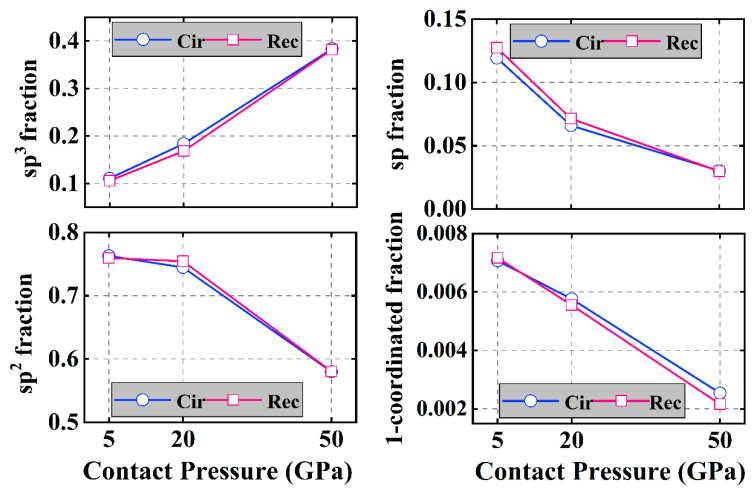
Changes in the fractions of sp^3^-C, sp^2^-C, sp-C, and 1-coordinated C hybridized bonds of friction systems with circle- and rectangle-textured shapes, respectively, under different contact pressures after sliding process.

To investigate possible friction mechanisms caused by the contact pressure and textured shape of a-C films, it is necessary to analyze the interfacial structural change that is closely related to the friction behavior [10]. Figure 7 shows the variation of the carbon hybridized structure with contact pressure for both the circular and rectangular textured systems after the sliding process. First, for the circular textured system, with the contact pressure increased from 5 to 50 GPa, the sp^3^ hybridized fraction increases in an obvious manner, which is followed by a decrease of sp^2^, sp, and 1-coordinated C fractions, suggesting a pressure-induced structural transformation of sp-to-sp^3^ and sp^2^-to-sp^3^. Due to the sufficient passivation of the interface and the reduction of the suspension bond, the increase in friction force is less than the increase rate of the normal load, resulting in the friction coefficient being reduced with the increase of contact pressure, as shown in Figure 3. A similar behavior is also observed in the rectangular textured system.

However, it is noted that for these two textured shapes, at a low contact pressure, the sp^3^ fraction of the circular textured system is higher than that of the rectangular textured case, while the content of unsaturated bonds, especially the sp hybridized fraction, is smaller, which is the main reason for the small friction coefficient. With the increase of high contact pressure, the circular and rectangular textured systems exhibit similar hybridized fraction values, resulting in a similar friction coefficient value (Figure 3).

**Figure 8:**
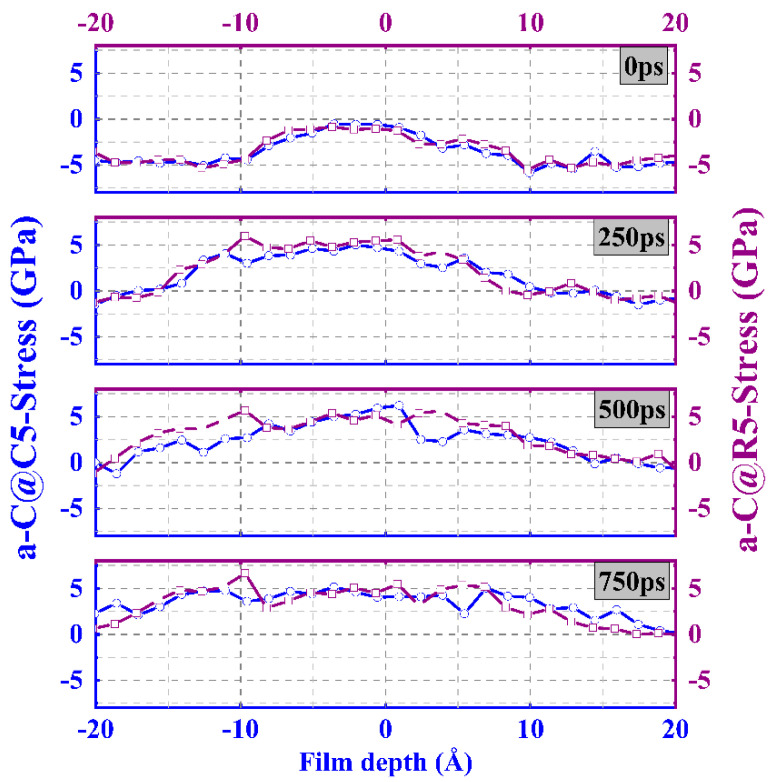
Stress distribution of a-C@C5 and a-C@R5 systems, respectively.

In order to explain the change in the structural transformation, Figure 8 and Figure S4 of the Supplementary Data further plot the stress curves of each system. It can be seen that the trends in the distribution of stress detected in both the a-C@C5 system and the a-C@R5 system (Figure 8) are similar; with the increase of sliding time, the tensile stress increases gradually, which is due to the cross-linking of the interface, leading to the stretching, breaking, and re-forming of the chemical bond in the process of shear sliding. It is worth noting that the increase in tensile stress in the a-C@R5 system is slightly higher than that in the a-C@C5 system, which implies a lower sp^3^ content according to the *P*-*T* phase diagram [27]. Considering the rise in contact pressure, the interfacial stress changes from tensile stress to compressive stress, resulting in a substantial rise in the sp^3^ content. This is in keeping with the results of the hybridized structure changes shown in Figure 7. After conducting a quantitative analysis of the variations in contact pressure and residual stress (Figure S5 of the Supplementary Data), we discover that when increasing the contact pressure, the residual stress also gradually rises, with the stress state changing from tensile to compressive stress, ultimately resulting in a rise in the sp^3^ content, consistent with previous reports [25,28].

## 4. Conclusions

In this research, the relationship between the tribological characteristics and the textured forms of a-C films under various contact pressures was investigated using RMD modeling, and the main goal of the analysis of the interfacial structure was to clarify the underlying friction mechanism. The following was revealed by the results:

1. The tribological properties of a-C films were sensitive to the textured shape under low contact pressure, and the tribological properties of the circular textured system were better than those of the rectangular textured system. This was due to the higher fraction of the sp^3^ hybridized structure and smaller unsaturated bond fractions at the friction interface.
2. When increasing the contact pressure from 5 to 50 GPa, on the one hand, the stress at the friction interface changed from a tensile to compressive state following the significant sp-to-sp^3^ and sp^2^-to-sp^3^ transformation. This not only induced the passivation of the friction interface, but also decreased the content of dangling bonds in an obvious manner, which led to the growth rate of friction forces being slower than that of the normal load, accounting for the significant decrease in the friction coefficient with contact pressure. However, on the other hand, under such high contact pressure, these two textured systems exhibited similar changes in the interfacial structure, including the hybridized structure and the interfacial stress, so no obvious difference in the friction coefficient was observed. These findings contribute to the in-depth investigation of the friction mechanism of textured a-C films, and, most importantly, they can guide scientific and technical applications for the creation of innovative and effective carbon-based lubrication systems.

## Data Availability

Not applicable.

## Supplementary Materials

The following supporting information can be downloaded at: https://www.mdpi.com/article/10.3390/ma16186108/s1.
