# Supplementary material for: Atomic-Scale Understanding on the Tribological Behavior of Amorphous Carbon Films under Different Contact Pressures and Surface Textured Shapes
Source: Materials (Basel). 2023 Sep 7;16(18):6108. doi: 10.3390/ma16186108 (PMC10532578; doi:10.3390/ma16186108)
Supplement: Supplementary file 1 [file materials-16-06108-s001.zip › materials-2555678-supplementary.pdf]

Supplementary Data

Atomic-scale Understanding on the Tribological Behavior of Amorphous Carbon Films under Different Contact Pressures and Surface Textured Shapes

Zan Chen,[1] Naizhou Du,[1] Xiaowei Li,[1,2*] Xubing Wei,[1] Jiaqing Ding,[1] Shiqi Lu,[1] Shuangjiang Du,[1] Cunao Feng,[1] Kai Chen,[1] Dekun Zhang,[1] Kwang-Ryeol Lee[3*]

[1] School of Materials Science and Physics, China University of Mining and Technology, Xuzhou 221116, China

[2] Key Laboratory of Marine Materials and Related Technologies, Zhejiang Key Laboratory of Marine Materials and Protective Technologies, Ningbo Institute of Materials Technology and Engineering, Chinese Academy of Sciences, Ningbo 315201, China

[3] Computational Science Center, Korea Institute of Science and Technology, Seoul 136-791, Republic of Korea

* Corresponding authors. E-mail address: lixw0826@gmail.com, xwli@cumt.edu.cn (X. Li)
E-mail address: krlee@kist.re.kr (K.R. Lee)

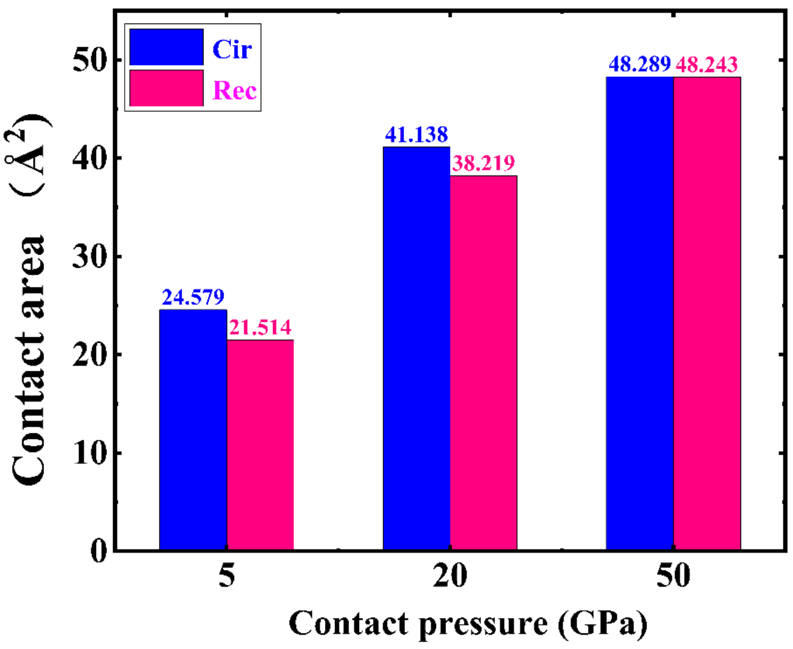

Figure S1. Contact area of friction systems with circle- and rectangle-textured shapes, respectively, under different contact pressures.

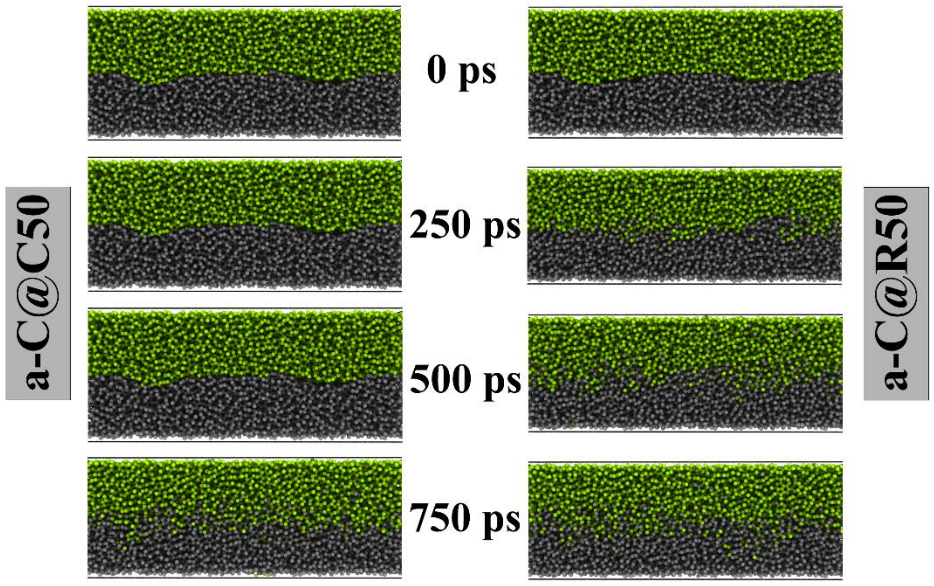

Figure S2. Evolutions of interfacial morphologies of a-C@C50 and a-C@R50 systems, respectively, under the contact pressure of 50 GPa.

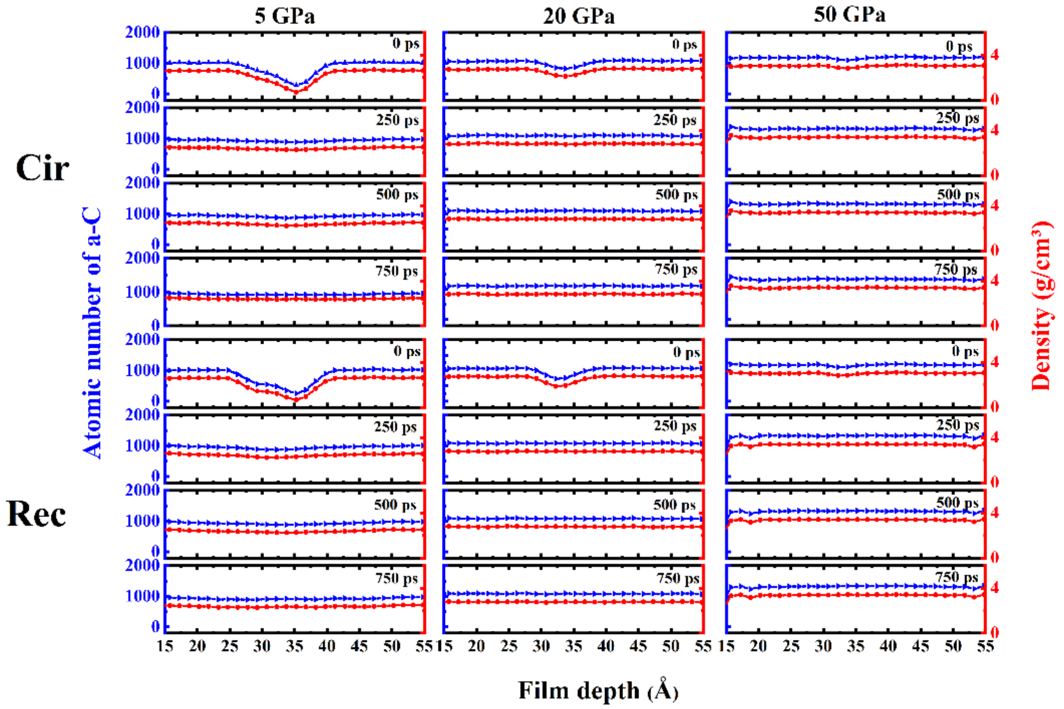

Figure S3. Atomic number and density distributions of friction systems with circle- and rectangle-textured shapes, respectively, under different contact pressures.

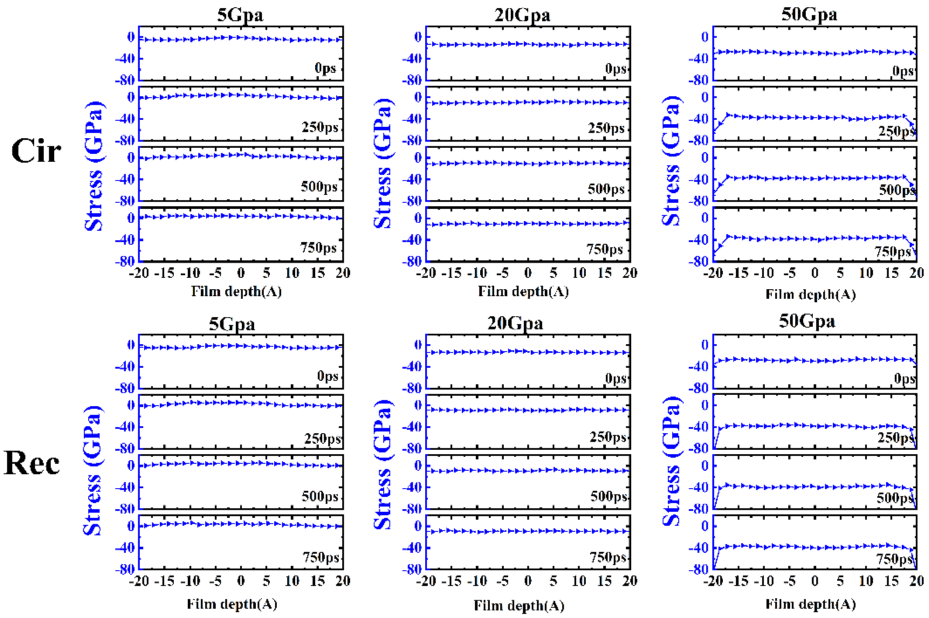

Figure S4. Stress distributions of friction systems with circle- and rectangle-textured

shapes, respectively, under different contact pressures.

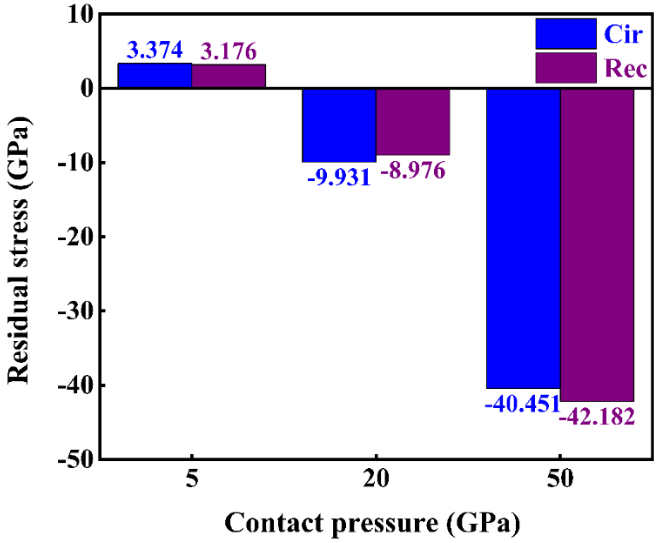

Figure S5. Residual stress of friction systems with circle- and rectangle-textured shapes, respectively, under different contact pressures.
